# Photonics Gets a Makeover: The New Era of Perovskite Devices

**DOI:** 10.3390/mi16070832

**Published:** 2025-07-21

**Authors:** Muhammad Danang Birowosuto

**Affiliations:** Łukasiewicz Research Network—PORT Polish Center for Technology Development, Stabłowicka 147, 54-066 Wrocław, Poland; muhammad.birowosuto@port.lukasiewicz.gov.pl

The story of perovskite materials dates back over a century to the discovery of calcium titanate, known for its nearly cubic crystal structure [1]. Originally valued for their dielectric properties in capacitors and transducers [2], perovskites have since found extensive utility in photonic applications due to their remarkable structural and chemical versatility [3,4].

Around five decades ago, interest in all-inorganic perovskites (AIPs) surged, leading to the development of yttrium aluminum perovskites as laser gain media [5]. Two decades later, hybrid organic–inorganic perovskites (HOIPs) emerged as promising candidates for light-emitting devices, initiated by foundational work at T.J. Watson Research Center [6]. Perovskites were, soon after, investigated as scintillators for radiation detection [7,8]. This rich evolution underscores the adaptability of the material system across diverse photonic technologies [9,10].

In recent years, perovskite solar cells (PSCs) have reignited widespread interest. Since their first demonstration in 2009 with a modest power conversion efficiency (PCE) of 3% [11], PSCs have undergone remarkable progress, now achieving a certified PCE of 26.95% [12,13]. To compete with silicon, perovskites must combine high efficiency with long-term operational stability, ideally exceeding 25 years. Their tunable compositions and superior optoelectronic properties make them highly competitive candidates [14].

Beyond photovoltaics, perovskites are gaining traction in emerging photonic systems. One particularly promising direction involves terahertz-frequency light-based data transmission, critical for ultrafast communication technologies supporting next-generation computing and the Internet of Things [15,16].

This editorial highlights recent key contributions published in *Micromachines*, contextualized within broader technological trends. The focus is organized around three main themes: nonlinear photonic properties [17,18], solar cell integration [12,13,19], and applications in photodetectors [20,21] and light-emitting diodes [22,23] (Figure 1).

For nonlinear photonics, Hardhienata et al. [17] explored the second harmonic generation (SHG) in tetragonal ABO_3_ perovskites using a nonlinear bond model. They showed how symmetry, light polarization, and spatial dispersion affect SHG, offering tunability via engineered polarizations. Their discussion of the Rashba effect and recombination processes has implications for both nonlinear optics and HOIP-based photovoltaics. More recently, Kowal et al. [18] demonstrated plasmonic-photonic heterostructures incorporating perovskite films. Simulations revealed strong field confinement and nonlinear enhancement at metal/perovskite interfaces, particularly in low-bandgap systems. These studies illustrate the evolution from theory to functional nonlinear platforms [24,25,26].

PSCs have rapidly evolved from conventional lead-based compositions to more environmentally benign alternatives. Seyed-Talebi et al. [19] investigated CsSnI_3_-based AIP solar cells, analyzing different hole transport layers (HTLs) and back-contact materials. They identified Cu_2_ZnSnSe_4_ as the most effective HTL, achieving a PCE of 21.63%—a notable figure close to the highest certified perovskite efficiency of 26.95% [12,13]. This underscores the practical viability of lead-free perovskite technologies and the alignment of *Micromachines* with leading-edge solar research.

Perovskite photodetectors (PDs) have emerged as leading candidates for high-speed optoelectronics. Wang et al. [21] reported a self-powered UV PD based on Y_2_O_3_-doped CuSCN and CsPbBr_3_, delivering a responsivity of 534 mA W^−1^ and an on/off ratio of 2.47 × 10^6^, with rapid switching (rise: 9 ms, fall: 5 ms). Complementing this, Sun et al. [20] introduced a self-powered polarization-sensitive PD using a buried grating structure fabricated by ultra-fast laser direct writing. This architecture obviates the need for external power or polarization optics and achieves sub-2 μs response times and 403 mA W^−1^ responsivity, among the fastest for PSPPDs. Both devices retained performance after extended air exposure, signaling real-world readiness.

Perovskite light-emitting diodes (PeLEDs) have also benefited from interfacial and crystallization control. In 2025, Chen et al. [22] used a two-step solid-state diffusion process with thermal crystallization to modulate PbBr_2_ and CsBr diffusion, yielding CsPbBr_3_ films with dense morphology and a current efficiency of 7.1 cd/A, a 200% enhancement over conventional methods. This performance approaches the state-of-the-art CsPbBr_3_ PeLED of 10.3 cd/A [23], reflecting tangible progress in PeLED engineering.

In conclusion, perovskite materials have transformed from structural curiosities into essential components of modern photonic technologies. Their structural tunability and superior optoelectronic performance continue to unlock new applications in energy harvesting, ultra-fast communications, sensing, and light emission. The works highlighted in this editorial exemplify not just incremental improvements but a paradigm shift in how perovskites are utilized, demonstrating that this new era of perovskite devices is not just a makeover, but a transformation.

## Figures and Tables

**Figure 1:**
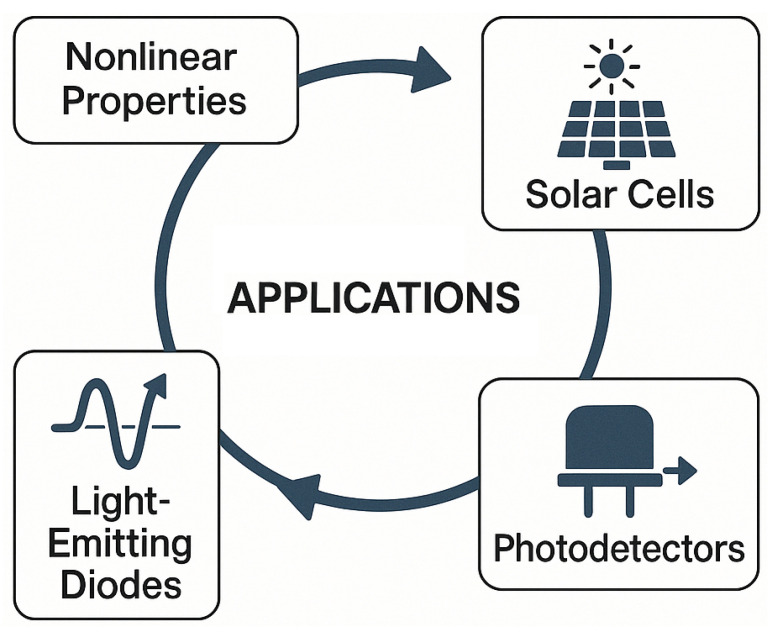
Overview of recent advances in perovskite photonic devices.
